# Arteriolar hyalinosis and renal outcomes in patients with immunoglobulin A nephropathy

**DOI:** 10.1080/0886022X.2022.2083974

**Published:** 2022-06-06

**Authors:** Yunzhu Shen, Tangli Xiao, ZhiKai Yu, Yinghui Huang, Ting He, Haiyang Li, Jun Zhang, Jiachuan Xiong, Jinghong Zhao

**Affiliations:** Department of Nephrology, the Key Laboratory for the Prevention and Treatment of Chronic Kidney Disease of Chongqing, Chongqing Clinical Research Center of Kidney and Urology Diseases, Xinqiao Hospital, Army Medical University (Third Military Medical University), Chongqing, P.R. China

**Keywords:** Immunoglobulin A nephropathy, arteriolar hyalinosis, hypertension, renal outcomes

## Abstract

**Background:**

The relationship between arteriolar hyalinosis and renal progression in immunoglobulin A nephropathy (IgAN) is not fully understood. We aimed to investigate the clinicopathological features and outcomes of IgAN with or without arteriolar hyalinosis.

**Methods:**

A total of 762 diagnosed with IgAN patients were retrospectively analyzed. We classified IgAN patients into two groups with or without arteriolar hyalinosis. Then, the clinicopathological characteristics of the two groups were compared. We used Kaplan–Meier survival analysis to compare the composite kidney outcome of the two groups and applied multivariate Cox regression analyses to test the association between arteriolar hyalinosis and composite kidney outcome.

**Results:**

Overall, 412 (54.1%) patients had arteriolar hyalinosis, including 173 patients diagnosed with hypertension. IgAN patients with arteriolar hyalinosis were older and had higher proteinuria, urea, uric acid, and blood pressure, while lower eGFR than those without arteriolar hyalinosis. Subgroup analysis showed similar results in IgAN patients with hypertension. Kaplan–Meier survival analysis showed that IgAN patients with arteriolar hyalinosis had worse composite kidney outcome than those without arteriolar hyalinosis. In addition, subgroup analysis revealed that patients with hypertension have worse composite kidney outcome than those without hypertension. Multivariate Cox regression analyses confirm that arteriolar hyalinosis (HR 2.57; 95% CI 1.41–4.69; *p* = 0.002) is an independent risk factor for renal prognosis in IgAN patients.

**Conclusions:**

Our study demonstrated that arteriolar hyalinosis is a common vascular lesion in IgAN patients. Arteriolar hyalinosis connects closely with hypertension, and arteriolar hyalinosis is an independent risk factor for renal prognosis in patients with IgAN.

## Introduction

Immunoglobulin A nephropathy (IgAN) is the most common primary glomerular nephropathy globally [1]. A systematic review of biopsy-based studies in multiple countries suggests that the incidence of IgAN is at least 1.5 per 100 000 population [2]. A previous study has also confirmed that IgAN was the most common cause of chronic kidney disease (CKD) in China [3]. Although significant progress has been made in the treatment of IgAN, IgAN has still been reported to reduce life expectancy by more than 10 years and cause kidney failure in 20–40% of patients within 20 years of diagnosis. Furthermore, the disease has a tremendous economic impact and represents a social burden [4]. And meanwhile, the prognosis of individuals with IgAN is exceptionally heterogeneous.

It is well-known that the clinical features of IgAN mainly include proteinuria, microscopic hematuria, high blood pressure (one or more symptoms) [5], And the IgA deposition is a typical pathological feature [6], Jan Novak et al. put forward a hypothesis that processing several hits would contribute to the formation of pathogenic immune complexes with galactose-deficient IgA1 as the key autoantigen [7]. Yet, the accurate diagnosis and classification of IgAN mainly depend on the renal biopsy [8]. The pathological classifications go through three major versions, including Lee’s classification, Haas classification, and the newest Oxford classification [9]. Although these classifications are useful in diagnosing and grading IgAN, we noted that the focus of these classifications is quite different. New diagnostic criteria also seek a more comprehensive description of the pathological lesions, but some pathological features, such as vascular lesions, are not included in the classification criteria [10,11].

In recent years, studies on the intrarenal vascular lesions of IgAN have attracted significant attention. For example, intrarenal vascular lesions, including arterial intimal thickening, arteriolar hyalinosis, and microangiopathy, are common in IgAN [12]. A previous study indicated that the prevalence of renal arteriole and arteriolar lesions was 54.6% in patients with IgAN [13]. And those vascular lesions are associated with renal failure [14]. Arteriolar hyalinosis is a kind of intrarenal vascular lesion, which is common in the renal pathological reports in IgAN [14,15]. However, it is uncertain whether arteriolar hyalinosis is related to progress and prognosis in patients with IgAN [13]. Therefore, the current study investigates the clinical, pathological characteristics, and renal outcomes in patients with IgAN with or without arteriolar hyalinosis.

## Material and methods

### Participants

A total of 762 patients with IgAN diagnosed by renal biopsy were admitted to the nephrology department, Xinqiao Hospital, Army Medical University between January 2012 and December 2018. These clinicopathological materials were retrospectively analyzed. This study was approved by the ethics committee of Xinqiao Hospital and adhered to the principles of the Declaration of Helsinki. Our study has obtained informed consent from all the patients.

### Inclusion and exclusion criteria

**Figure 1.**
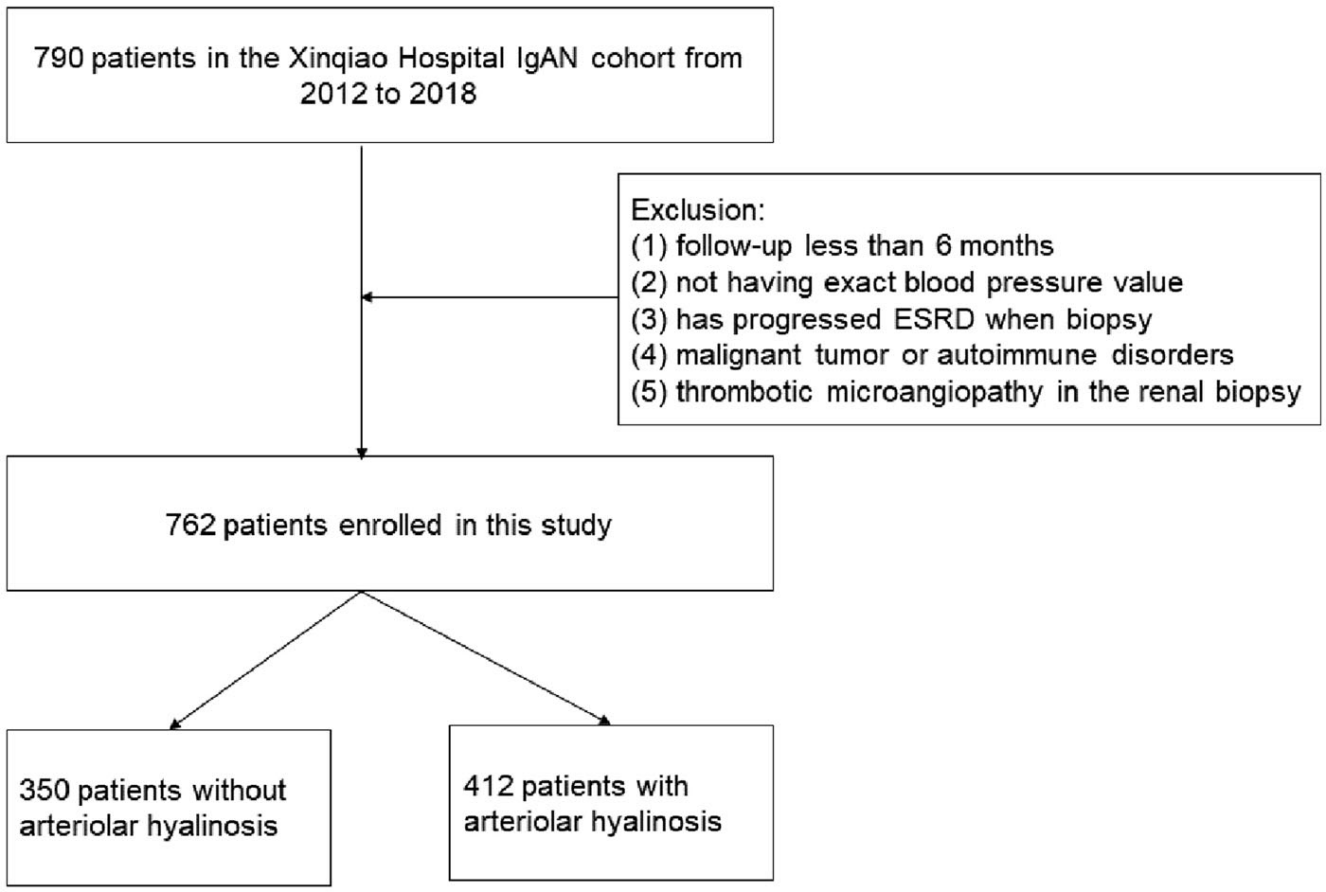
Flowchart showing the procedure for the selection of study participants with or without arteriolar hyalinosis in the patients of IgAN.

Patients with (a) diagnosed with IgAN by renal biopsy and (b) a regular medical history with follow-up data in Xinqiao Hospital (c) the number of glomeruli ≥ 8 were included. While patients with (a) follow-up less than 6 months, (b) no exact blood pressure value, (c) had already progressed to end-stage renal disease (ESRD), (d) diabetes or malignant tumors or other autoimmune disorders, (e) thrombotic microangiopathy in the renal biopsy were excluded. The flowchart is shown in Figure 1.

### Clinical data

We collected the clinical data of every patient at the time of renal biopsy. These data include sex, age, height, weight, blood pressure, and inflammation markers which include C-reactive protein (CRP) and tumor necrosis factor α (TNF-α), hemoglobin, platelet, serum complementary 3 (C3), serum complementary 4 (C4), serum albumin, urea, serum creatinine, serum urea acid, total cholesterol and total proteinuria of 24 h, and estimated glomerular filtration rate (eGFR) was calculated using the CKD epidemiology collaboration (CKD-EPI) equation [16]. Hypertension is defined as systolic blood pressure (SBP) ≥ 140 mmHg or diastolic blood pressure (DBP) ≥ 90 mmHg or taking antihypertensive medication.

### Pathological data and criteria of arteriolar hyalinosis

All renal biopsy specimens were processed routinely for immunofluorescence microscopy and light microscopy, Sections were stained for direct immunofluorescence with fluorescein isothiocyanate–conjugated antibodies specific for human IgA, IgG, IgM, C3, C4, and C1q. Immunofluorescence results were graded on a scale of 0 to 3, and a score ≥1 was regarded as immunofluorescence positive in this study. Sections used for light microscopy were stained with hematoxylin and eosin, periodic acid–Schiff, and Masson’s trichrome stain. Sections for light microscopy were graded according to the Oxford MEST-C score of IgAN [10]. Two pathologists (T.X. and J.Z.) who were blinded to clinical data reviewed kidney biopsies from all patients independently. All disagreements were finally resolved by the senior pathologist (H.S.).

Arteriolar hyalinosis consists of the deposition of hyaline material into the vascular wall coupled with matrix protein synthesis. Accumulation of a large amount of amorphous, eosinophilic, glassy material replaces the media and has resulted in thickening and rigidity of the wall and apparent attenuation of the lumen [15]. And in our study, we classified 0, 1–25, 26–50% according to the estimation of the fraction of most-affected arteriolar wall replaced by hyaline in one complete LM section [17].

### Outcomes

The primary outcome of interest was a composite kidney outcome defined as more than a 30% reduction in eGFR [18–20], or ESRD. The last follow-up time was 31 October 2020.

### Statistical analysis

All continuous variables were expressed as the mean ± standard deviation (normally distributed variables) or median with interquartile range (IQR: non-normally distributed variables) for data distribution. Enumeration data were expressed in percentages. The *t*-test was applied for normally distributed data, and the Mann-Whitney *U* test was applied for non-normally distributed data. The chi-squared test was applied for enumeration data. And the Kaplan–Meier method works to estimate renal survival time. A log-rank test was used to compare differences between renal survival curves. Univariate and multivariate Cox regression analyses were performed to test the association between arteriolar hyalinosis and the primary event of interest and reported hazard ratio (HR) with 95% confidence interval (CI) and *p* values. Multivariate Cox models included univariate, Model 1, Model 2, and Model 3. Model 1 included clinical variables such as age, sex, BMI, TNF-α, mean arterial pressure (MAP), proteinuria of 24 h, total cholesterol, uric acid, eGFR, and Model 2 is model 1 plus pathological variable such as global glomerulosclerosis, mesangial hypercellularity (M), endocapillary proliferation (E), segmental glomerulosclerosis (S) and tubular atrophy/interstitial fibrosis (T) and crescents (C), deposition of C3, deposition of C4. Model 3 is model 2 plus Renin-angiotensin-aldosterone System (RAAS) blockade, other immunosuppressants, and glucocorticoid. All *p* values were two-tailed, and values < 0.05 were considered statistically significant. The statistical analysis was performed by the SPSS statistics (version 25.0, IBM Corporation) and GraphPad Prism (version 8.0, GraphPad Software, Inc.).

## Results

### Characteristics of the cohort

**Figure 2.**
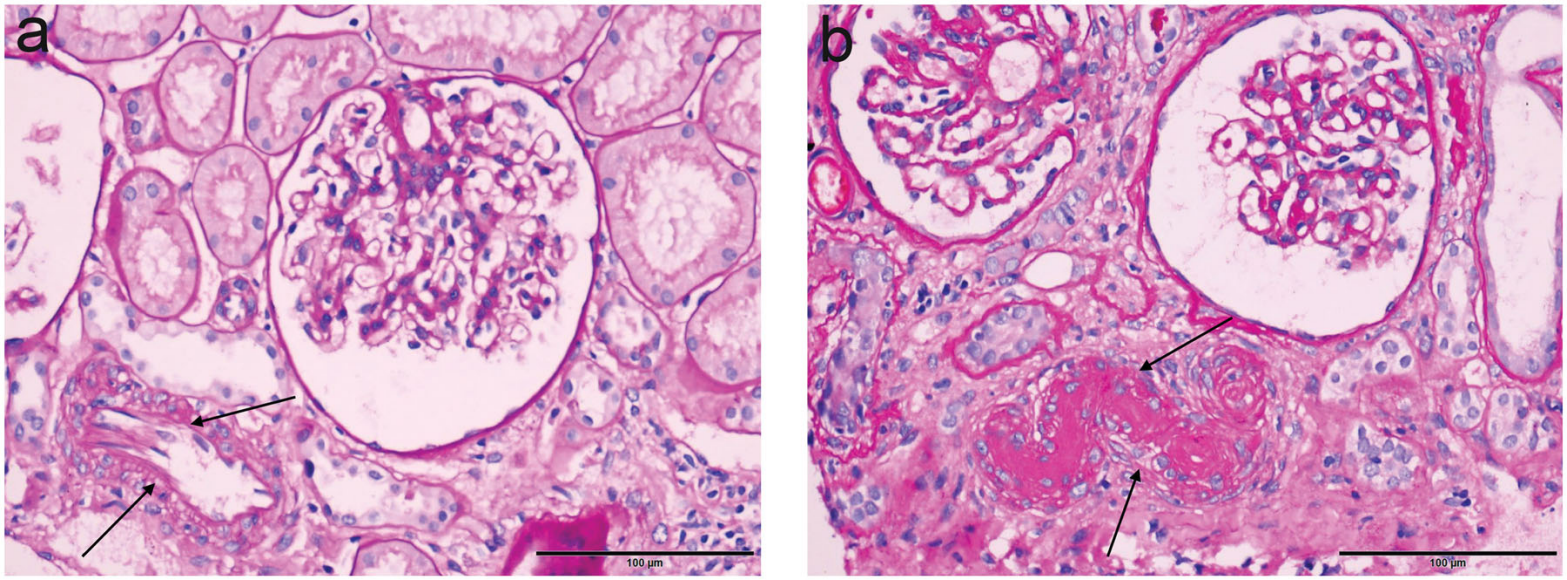
Typical examples in immunoglobulin A nephrology using light microscopy. (a) The presence of no-arteriolar hyalinosis, shown as there is no pink material around this artery; (b) The presence of arteriolar hyalinosis (arrow), shown as homogeneous pink hyaline material (arrow), is identified on the wall of this artery. (a, b: PAS stain). Scale bars represent 100 μm.

A total of 762 patients with IgAN were included; the essential characteristics are shown in Table 1. The mean age was 36.21 ± 11.07 years. Men accounted for 45.3% of the population, over one-third of the population has hypertension, proteinuria of 24 h was 0.65 (IQR 0.24–1.47) g/24 h, while the average serum creatinine was 86.05 μmol/L (IQR 65.68–112.83) and the median eGFR was 89.46 mL/min/1.73 m^2^ (IQR 60.97–110.93). In addition, over 50% of the patients were diagnosed with arteriolar hyalinosis under renal biopsy, and the representative images of arteriolar hyalinosis are shown in Figure 2. Then, the included patients were divided into IgAN with arteriolar hyalinosis group and IgAN without arteriolar hyalinosis group. Compared with IgAN without arteriolar hyalinosis group, patients in IgAN with arteriolar hyalinosis group were older (38.29 vs. 33.75, *p* < 0.001), had a higher proportion of hypertension (42.0 vs. 20.9%, *p* < 0.001), heavier proteinuria (0.77 vs. 0.56, *p* = 0.002), higher serum uric acid (394.60 vs. 364.20, *p* < 0.001), and higher total cholesterol (4.61 vs. 4.47, *p* = 0.004), while a lower level of eGFR (76.84 vs. 102.82, *p* < 0.001). Furthermore, renal biopsy findings showed a higher proportion of global glomerulosclerosis (9.5 vs. 4.3%, *p* < 0.001) and a higher deposition rate of C3 (89.1 vs. 82.9%, *p =* 0.013) in IgAN with the arteriolar hyalinosis group. Besides that, there were also significant differences in mesangial hypercellularity (90.0 vs. 78.0%, *p* < 0.001), segmental glomerulosclerosis (70.4 vs. 47.4%, *p* < 0.001), and tubular atrophy/interstitial fibrosis (29.4 vs. 3.7%, *p* < 0.001) between the two groups in the Oxford classification. Interestingly, our work found that more patients in IgAN with arteriolar hyalinosis were treated with glucocorticoid (51.7 vs. 43.1%, *p* = 0.018) and immunosuppressants (16.5 vs. 11.4%, *p* = 0.045).

**Table 1.** Analysis of no-arteriolar hyalinosis and arteriolar hyalinosis.

Characteristic	Total analysis	No arteriolar hyalinosis	Arteriolar hyalinosis	*p*-value
Patient, No	762	350	412	
Follow up, month	34 (26–45)	37 (26–56)	32 (25–41)	<0.001
Age (years)	36.21 ± 11.07	33.75 ± 10.42	38.29 ± 11.18	<0.001
Women, *n* (%)	417 (54.7%)	196 (56.0%)	221 (53.6%)	0.514
BMI (kg/m^2^)	22.98 ± 3.30	22.78 ± 3.53	23.15 ± 3.09	0.125
Hypertension	246 (32.3%)	73 (20.9%)	173 (42.0%)	<0.001
SBP (mmHg)	125.71 ± 18.90	121.23 ± 17.23	129.51 ± 19.43	<0.001
DBP (mmHg)	81.52 ± 13.44	78.76 ± 13.43	83.87 ± 13.02	<0.001
CRP (mg/L)	2.50 (1.90–3.80)	2.50 (1.90–3.80)	2.50 (1.90–3.80)	0.804
TNF-α (pg/mL)	8.00 (6.58–9.90)	8.00 (6.20–9.60)	8.00 (6.70–10.30)	0.008
C3 (g/L)	0.85 ± 0.18	0.85 ± 0.19	0.85 ± 0.18	0.871
C4 (mg/dL)	19.40 (16.20–22.30)	19.40 (16.08–21.65)	19.40 (16.20–22.70)	0.048
HB (g/L)	129.11 ± 19.25	129.69 ± 18.67	128.62 ± 19.74	0.445
PLT (10^9^/L)	218.45 ± 66.04	217.03 ± 66.57	219.67 ± 65.63	0.583
ALB (g/L)	40.88 ± 6.46	40.83 ± 6.98	40.92 ± 5.99	0.857
Urea (mmol/L)	5.48 (4.35–6.98)	5.09 (4.12–6.27)	5.78 (4.61–7.54)	<0.001
Proteinuria (g/24h)	0.65 (0.24–1.47)	0.56 (0.21–1.25)	0.77 (0.28–1.66)	0.002
Scr (μmol/L)	86.05 (65.68–112.83)	78.50 (61.33–96.20)	93.20 (72.10–125.20)	<0.001
eGFR (mL/min/1.73 m^2^)	89.46 (60.97–110.93)	102.82 (78.25–116.63)	76.84 (52.83–101.66)	<0.001
Uric acid (μmol/L)	380.64 ± 101.21	364.20 ± 92.64	394.60 ± 106.08	<0.001
Cholesterol (mmol/L)	4.55 (3.96–5.21)	4.47 (3.82–5.09)	4.61 (4.10–5.28)	0.004
Renal biopsy				
Total glomeruli	14.00 (11.00–18.00)	13.00 (10.75–17.25)	14.00 (11.00–18.00)	0.191
Global glomerulosclerosis (%)	7.1 (0.0–20.0)	4.3 (0.0–13.6)	9.5 (0.0–27.0)	<0.001
Oxford classification				
M1	644 (84.5%)	273 (78.0%)	371 (90.0%)	<0.001
E1	26 (3.4%)	14 (4.0%)	12 (2.9%)	0.410
S1	456 (59.8%)	166 (47.4%)	290 (70.4%)	<0.001
T1/T2	134 (17.7%)	13 (3.7%)	121 (29.4%)	<0.001
C1/C2	144 (18.9%)	64 (18.3%)	80 (19.4%)	0.691
IF positive				
C3	657 (86.2%)	290 (82.9%)	367 (89.1%)	0.013
C4	5 (0.7%)	1 (0.3%)	4 (1.0%)	0.382
Medications				
RAAS blockade	628 (82.4%)	285 (81.4%)	343 (83.3%)	0.510
Immunosuppressant	108 (14.2%)	40 (11.4%)	68 (16.5%)	0.045
Glucocorticoid	364 (47.8%)	151 (43.1%)	213 (51.7%)	0.018
Outcome				
Composite kidney outcome	87 (11.4%)	30 (8.6%)	57 (13.8%)	0.023

RAAS blockade includes angiotensin-converting enzyme inhibitors and angiotensin receptor blockers; composite kidney events including >30% reduction in estimated glomerular filtration rate, or end-stage renal disease.

Abbreviations: BMI: Body mass index; Scr: Serum creatinine; eGFR, estimated glomerular filtration rate.

### Arteriolar hyalinosis and composite kidney outcome

**Figure 3.**
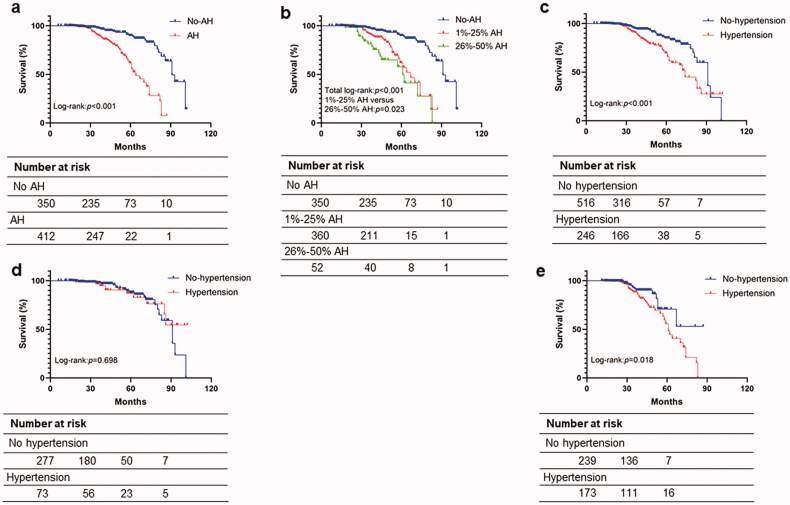
Survival from the composite kidney outcome of >30% reduction in estimated glomerular filtration rate, end-stage renal disease. *p* values for differences in survival were calculated using the log-rank test. Survival analysis of (a) presence or absence of arteriolar hyalinosis, (b) different degree of arteriolar hyalinosis, (c) presence or absence of hypertension, (d) without or with hypertension subgroup analysis of no-arteriolar hyalinosis, (e) without or with hypertension subgroup analysis of arteriolar hyalinosis. AH: arteriolar hyalinosis.

During the follow-up of 2.8 years, 30 (8.6%) patients in IgAN without arteriolar hyalinosis group and 57 (13.8%) patients in IgAN with arteriolar hyalinosis group reached the composite kidney outcome. Kaplan-Meier curve analysis showed survival rate in IgAN with arteriolar hyalinosis was significantly lower than that of patients without arteriolar hyalinosis (log-rank test, *p* < 0.001; Figure 3(a)), and survival rates in different degrees of arteriolar hyalinosis were also being analyzed (total log-rank test, *p* < 0.001; Figure 3(b)). Kaplan–Meier analysis also revealed a significant difference in the renal survival between patients with hypertension and without hypertension (log-rank test, *p* < 0.001; Figure 3(c)). Then, univariate and multivariate Cox proportional hazards regression models were generated. In the crude model, patients with arteriolar hyalinosis had increased near fourfold risk of renal outcomes compared with patients without arteriolar hyalinosis, after adjusting for age, sex, BMI, TNF-α, mean arterial pressure, proteinuria of 24 h, glucocorticoid, RAAS blockade, immunosuppressants, total cholesterol, serum uric acid, eGFR, global glomerulosclerosis and Oxford classification (MEST-C scores), mesangial deposition of C3, mesangial deposition of C4 (Model 3), the arteriolar hyalinosis remains an independent risk factor for the progression of the composite kidney events (HR 2.57; 95% CI 1.41–4.69; *p* = 0.002). Furthermore, compared with patients without arteriolar hyalinosis, we found that patients with a higher ratio of arteriolar hyalinosis (26–50%) have a much higher risk of composite kidney outcome (HR 4.20; 95% CI 2.02–8.73; *p* < 0.001; Table 2).

**Table 2.** Cox regression models for the composite kidney outcome in IgAN patients.

	Univariate		Model 1		Model 2		Model 3	
	HR (95% CI)	*p* value	HR (95% CI)	*p* value	HR (95% CI)	*p* value	HR (95% CI)	*p* value
Arteriolar hyalinosis^a^								
Yes vs. no	4.41 (2.69–7.22)	<0.001	2.93 (1.70 − 5.03)	<0.001	2.62 (1.44–4.75)	0.002	2.57 (1.41–4.69)	0.002
1–25% vs. no	3.66 (2.16–6.24)	<0.001	2.58 (1.46–4.58)	0.001	2.24 (1.20–4.21)	0.012	2.15 (1.14–4.06)	0.018
26–50% vs. no	6.80 (3.70–12.48)	<0.001	4.19 (2.12–8.27)	<0.001	3.96 (1.92–8.17)	<0.001	4.20 (2.02–8.73)	<0.001

Note: Model 1: adjusted for age, sex, BMI, TNF-α, mean arterial pressure, proteinuria of 24 h, total cholesterol, serum uric acid, eGFR; Model 2: Model 1 plus global glomerulosclerosis, mesangial hypercellularity (M), endocapillary proliferation(E), segmental glomerulosclerosis (S), tubular atrophy/interstitial fibrosis (T), crescents (C), deposition of C3, deposition of C4; Model 3: Model 2 plus steroids, RAAS blockade, other immunosuppressants.

^a^No-arteriolar hyalinosis is the reference group.

Abbreviations: CI, confidence interval; HR, hazard ratio.

### Subgroup analysis by hypertension

Considering a significantly higher proportion of hypertension in patients with arteriolar hyalinosis, we performed a subgroup analysis based on patients who have arteriolar hyalinosis with hypertension or without hypertension. Of the 412 patients with arteriolar hyalinosis, 173 (42.0%) patients were diagnosed with hypertension. The clinicopathological features are listed in Table 3. The two groups showed significant differences in age, sex, BMI, CRP, TNF-α, and C3 between the two groups. Moreover, patients with hypertension had a higher proteinuria (0.99 vs. 0.59, *p* = 0.001), uric acid (425.79 vs. 372.02, *p* < 0.001), serum creatinine (116.10 vs. 83.90, *p* < 0.001), and cholesterol (4.74 vs. 4.55, *p* = 0.004) than those without hypertension. In addition, in the pathological aspects, we found that the patients with hypertension had a higher percentage of T1/T2 (41.0 vs. 20.9%, *p* < 0.001), global glomerulosclerosis (14.3 vs. 7.1%, *p* < 0.001), and 26–50% of arteriolar hyalinosis (24.9 vs. 6.3%, *p* < 0.001) but a lower proportion of C1/C2 (14.5 vs. 23.0%, *p* < 0.030) than patients without hypertension. Moreover, the Kaplan–Meier curve showed that the survival percentage of patients with hypertension was not significantly different from patients without hypertension in the no-arteriolar hyalinosis group (log-rank test, *p* = 0.698; Figure 3(d)), while the survival percentage of patients with hypertension was significantly lower than that of patients without hypertension in arteriolar hyalinosis group (log-rank test, *p* = 0.018; Figure 3(e)).

**Table 3.** Subgroup analysis of IgAN patients with arteriolar hyalinosis.

Characteristic	Arteriolar hyalinosis		*p* value
	Without hypertension	With hypertension	
Patient, No	239	173	
Follow up, month	31 (23–39)	34 (26–44)	0.005
Age (years)	36.10 ± 10.10	41.31 ± 11.91	<0.001
Women, *n* (%)	139 (58.2%)	82 (47.4%)	0.031
BMI (kg/m^2^)	22.53 ± 2.84	24.02 ± 3.21	<0.001
CRP (mg/L)	2.50 (1.70–3.30)	2.80 (2.10–4.40)	<0.001
TNF-α (pg/mL)	8.00 (6.30–9.30)	8.71 (7.70–11.20)	<0.001
C3 (g/L)	0.83 ± 0.16	0.89 ± 0.19	<0.001
C4 (mg/dL)	19.40 (16.00–22.50)	19.60 (16.65–23.00)	0.168
HB (g/L)	128.36 ± 18.45	128.98 ± 21.44	0.752
PLT (10^9^/L)	222.13 ± 68.43	216.26 ± 61.59	0.371
ALB (g/L)	41.33 ± 5.85	40.33 ± 6.15	0.092
Urea (mmol/L)	5.45 (4.33–6.74)	6.79 (5.11–8.40)	<0.001
Proteinuria (g/24h)	0.59 (0.26–1.34)	0.99 (0.34–2.04)	0.001
Scr (μmol/L)	83.90 (65.60–105.90)	116.10 (89.95–156.50)	<0.001
eGFR (mL/min/1.73 m^2^)	93.20 (65.10–109.50)	58.34 (41.81–83.47)	<0.001
Uric acid (μmol/L)	372.02 ± 96.72	425.79 ± 110.72	<0.001
Cholesterol (mmol/L)	4.55 (4.04–5.07)	4.74 (4.20–5.39)	0.004
Renal biopsy			
Total glomeruli	15.00 (12.00–20.00)	13.00 (10.00–16.00)	<0.001
Global glomerulosclerosis (%)	7.1 (0.0–20.0)	14.3 (0.0–33.3)	<0.001
Arteriolar hyalinosis			
1–25%	224 (93.7%)	130 (75.1%)	<0.001
26–50%	15 (6.3%)	43 (24.9%)	
Oxford classification			0.054
M1	221 (92.5%)	150 (86.7%)	
E1	6 (2.5%)	6 (3.5%)	0.568
S1	176 (73.6%)	114 (65.9%)	0.089
T1/T2	50 (20.9%)	71 (41.0%)	<0.001
C1/C2	55 (23.0%)	25 (14.5%)	0.030
IF positive			
C3	216 (90.4%)	151 87.3%)	0.320
C4	2 (0.8%)	2 (1.2%)	1.000
Treatment			
RAAS blockade	198 (82.8%)	145 (83.8%)	0.795
Immunosuppressant	34 (14.2%)	34 (19.7%)	0.143
Glucocorticoid	121 (50.6%)	92 (53.2%)	0.609
Outcome			
Composite kidney outcome	17(7.1%)	40(23.1%)	<0.001

RAAS blockade includes angiotensin-converting enzyme inhibitors and angiotensin receptor blockers; composite kidney events including >30% reduction in estimated glomerular filtration rate, or end-stage kidney disease.

Abbreviations: BMI, Body mass index; Scr, Serum creatinine; eGFR, estimated glomerular filtration rate.

As shown in Table 4, in univariate and multivariate Cox proportional hazards regression models and the univariate Cox regression model indicated the hypertension was a risk factor for the progression of the composite kidney events (HR 1.93; 95% CI 1.08–3.44; *p* = 0.027), but after adjusting for age, sex, BMI, TNF-α, proteinuria, total cholesterol, uric acid, eGFR, global glomerulosclerosis, MEST-C scores, mesangial deposition of C3, mesangial deposition of C4, RAAS blockade, other immunosuppressants and glucocorticoid (Model 3), the outcome suggests that the hypertension is not the independent risk factor for the progression of the composite kidney event in the cohort of arteriolar hyalinosis patients (HR 1.82; 95% CI 0.91–3.63; *p* = 0.091).

**Table 4.** Cox regression models for the composite kidney outcome in IgAN patients of arteriolar hyalinosis with or without hypertension.

Model	HR (95%CI)		*p* value
	Without hypertension	With hypertension	
Univariate	Reference	1.93 (1.08–3.44)	0.027
Model 1		1.32 (0.68–2.55)	0.412
Model 2		1.51 (0.76–3.00)	0.234
Model 3		1.82 (0.91–3.63)	0.091

Note: Model 1: adjusted for age, sex, BMI, TNF-α, proteinuria of 24 h, total cholesterol, serum uric acid, eGFR; Model 2: Model 1 plus global glomerulosclerosis, mesangial hypercellularity (M), endocapillary proliferation(E), segmental glomerulosclerosis (S), tubular atrophy/interstitial fibrosis (T), crescents (C), deposition of C3, deposition of C4; Model 3: Model 2 plus steroids, RAAS blockade, other immunosuppressants.

Abbreviations: CI, confidence interval; HR, hazard ratio.

## Discussion

In the current study, we found that more than half of patients with IgAN have arteriolar hyalinosis. Patients with arteriolar hyalinosis have poor renal outcomes than patients without arteriolar hyalinosis, supporting that arteriolar hyalinosis is an independent predictor of the prognosis of IgAN patients.

The pathologic features of IgAN were complicated and extensive. Some just manifested like minimal change disease, some showed the focal segmental glomerular sclerosis, but some yet had mild mesangial proliferation and immunofluorescence indicated the deposition of IgA [21–23]. At present, the primary mechanism of the newest Oxford classification focuses on the renal tubule and renal interstitial changes [11]. However, some other changes may always be ignored in the pathological features, and it is not clear that these changes played in the progression and prognosis of IgAN.

Meanwhile, intrarenal vascular lesions were common in pathological reports of renal biopsy [13,14]. As we know, intrarenal vascular lesions can also be observed in several glomerular diseases, including acute kidney injury, CKD, lupus nephritis, diabetic nephropathy, hypertensive nephropathy, and so on [24–27]. Furthermore, intrarenal vascular lesions incorporated arterial lesions, arteriolar lesions, and microangiopathic lesions, and different types of lesions were often observed in the various pathological patterns. For example, acute lesions including endotheliocyte swelling and arteriolar thrombosis have been used in an evaluation system in lupus nephritis [26], arteriolar hyalinosis can be observed in IgAN, diabetic nephropathy [17]. Therefore, as we know, some intrarenal arterial lesions were common in most pathological patterns.

Furthermore, intrarenal vascular lesions remain to be shared in IgAN. Some studies have reported that the manifestations of vascular lesions are arterial wall fibrotic intimal thickening, arteriolar hyalinosis, arteriolar endotheliocyte swelling, arteriolar inflammatory cell infiltration, and arteriolar thrombosis in IgAN. The risk factors of these lesions are associations with hypertension, higher serum creatinine and uric acid, high urinary protein excretion, global glomerulosclerosis, tubular atrophy, and interstitial fibrosis [13]. And in the histopathologic diagnosis of renal biopsy, it is reported that the prevalence of intrarenal arterial lesions was up to 72.2% in IgAN [14]. And Cai et al. [12] reported about one-fifth (20.7%) of patients had arteriolar hyalinosis in their study. Meanwhile, it is reported that these lesions would cause poor renal outcomes in patients with IgAN [14,28]. Nevertheless, arteriolar hyalinosis is one of the most common lesions. It is always found in terminal interlobular arteries and proximal afferent arterioles, but the relationship with prognosis is uncertain in IgAN.

Our study confirmed significant differences in clinical and pathological features between the patients with arteriolar hyalinosis and without arteriolar hyalinosis in IgAN. Myllymaki et al. [29] analyzed the correlation between the serum uric acid and histopathological parameters and found that serum uric acid correlated significantly positive with arteriolar hyalinosis. What’s more, Feig et al. [30] said the mechanism of injury appears to be related to the development of the preglomerular arteriolar disease that impairs the renal autoregulatory response.

And univariate analysis [13] showed that high serum cholesterol was closely correlated with intrarenal arterial lesions. Chen et al. [31] reported that high C3 concentrations in plasma are associated with the development of hypertension. Bi et al. [32] identified serum C4 as a useful predictor for the prognosis of patients with IgAN. Wu et al. [33] analyzed a high intensity of C3 deposition predicts long-term poor renal survival for IgAN patients. Yang et al. [34] found Glomerular mesangial C4 deposition is independent predictor for poor prognosis. And this study has clarified that these items including serum uric acid, cholesterol, C4, and deposition of C3 are apparently higher in patients with IgAN with arteriolar hyalinosis than those without. In the arteriolar hyalinosis subgroup, the C3 and C4 concentration levels and intensity of C3 deposition are obviously higher in patients with hypertension than those without. The outcomes are consistent with the above studies.

Meanwhile, there are striking differences between the prognosis of the two groups, which implies that arteriolar hyalinosis is a pathological feature of poor prognosis. Furthermore, as the degree of arteriolar hyalinosis increases, the prognosis worsens. In addition, we have observed the relationship between arteriolar hyalinosis and hypertension. Wang et al. [35] analyzed that hypertensive IgAN patients with ischemic renal injury has a poorer prognosis. And this study has showed that the patients with arteriolar hyalinosis and hypertension in IgAN suffer a worse prognosis. And at the same time, hypertension seemed not to be an independent risk factor to the patients with arteriolar hyalinosis in this study, which is inconsistent with the study of Miyabe et al. [36]. The possible reasons are that pathological scoring systems for IgAN, such as Oxford classification, without considering arteriolar hyalinosis, and the MESTC primary are also included, while arteriolar hyalinosis is not included in multivariate regression analysis. Furthermore, the primary pathological manifestation of renal damage caused by the essential hypertension is arteriolar hyalinosis [37]. The possible reason for the results of our subgroup is that part of the effect of hypertension on the prognosis of IgAN works by causing hyaline changes in renal arterioles. However, it still needs further research on the internal mechanism of hypertension and arteriolar hyalinosis in IgAN.

A few limitations are able to make bias between the fact and our study. The first is that this was a retrospective study from the single-center, with limited sample capacity and a not too long follow-up period. But at the same time, the end event of our research is a composite kidney event. Therefore, our outcome needs to be proved by more extensive samples in multicenter by longer follow-up time. Second, we have just classified arteriolar hyalinosis into three degrees according to the relationship between the extent of deposits and the vessel wall diameter, but we have not calculated the index of arteriolar hyalinosis and not collected the data of fundoscopy or echocardiography. Above all may lead to a little deviation between this study and those of the public. These points need to be further evaluated in subsequent studies.

In conclusion, our results indicated that a large proportion of IgAN patients have arteriolar hyalinosis, which is an independent risk factor for renal progression. And the higher the degree of affected arterioles is, the greater the risk of worse renal outcomes will be. Examination of arteriolar hyalinosis for IgAN patients under renal biopsy is essential to renal pathologists and clinicians.
